# A criterion for sequential Cohen-Macaulayness

**DOI:** 10.1007/s00013-024-02011-y

**Published:** 2024-07-18

**Authors:** Giulio Caviglia, Alessandro De Stefani

**Affiliations:** 1 Department of Mathematics, Purdue University, 150 N. University Street, West Lafayette, IN 47907-2067 USA; 2 Dipartimento di Matematica, Università di Genova, Via Dodecaneso 35, Genoa, 16146 Italy

**Keywords:** Sequentially Cohen-Macaulay module, Arithmetic degree, Revlex order, 13D45, 13P10

## Abstract

The purpose of this note is to show that a finitely generated graded module *M* over $S=k[x_1,\ldots ,x_n]$, *k* a field, is sequentially Cohen-Macaulay if and only if its arithmetic degree ${\text {adeg}}(M)$ agrees with ${\text {adeg}}(F/{\text {gin}}_\textrm{revlex}(U))$, where *F* is a graded free *S*-module and $M \cong F/U$. This answers positively a conjecture of Lu and Yu from 2016.

## Introduction

Sequentially Cohen-Macaulay modules were first introduced in the graded setting by Stanley [20], in connection with the theory of Stanley-Reisner rings. Later, Schenzel [19] introduced the notion of Cohen-Macaulay filtered module in a more general setting, and showed that in the graded case it coincides with the one introduced by Stanley.

Let *k* be a field, and $S=k[x_1,\ldots ,x_n]$ with the standard grading. Let *M* be a finitely generated $\mathbb {Z}$-graded *S*-module of Krull dimension *d*. A module *M* is sequentially Cohen-Macaulay if one of the following three equivalent conditions is satisfied:

1. (Stanley) There exists a filtration $0 = M_0 \subsetneq M_1 \subsetneq M_2 \subsetneq \cdots \subsetneq M_t = M$ with $M_i/M_{i-1}$ Cohen-Macaulay graded *S*-modules of dimension $d_i$ satisfying $d_i>d_{i-1}$ for all $i=1,\ldots ,t$.
2. (Schenzel) If $\delta _i(M)$ denotes the largest submodule of *M* of dimension less than or equal to *i*, then the filtration
  $$\begin{aligned} 0 \subseteq \delta _0(M) \subseteq \delta _1(M) \subseteq \cdots \subseteq \delta _{d}(M) = M \end{aligned}$$
  is such that $\delta _i(M)/\delta _{i-1}(M)$ is either zero or Cohen-Macaulay for all $i=1,\ldots ,d$.
3. (Peskine) For every $i=0,\ldots ,n$, the module ${\text {Ext}}^{n-i}_S(M,S)$ is either zero or Cohen-Macaulay of dimension *i*.

Over the years, sequentially Cohen-Macaulay modules have been studied extensively, even from very different perspectives (for instance, see [1, 2, 7–9, 11–14]). We invite the interested reader to consult [6] for further details.

For the purposes of this note, it is important to mention a result of Herzog and Sbarra, which characterizes sequentially Cohen-Macaulay modules. We state it here in a dual version:

### Theorem 1.1

([15, Main theorem]). Let *F* be a finitely generated free *S*-module, and $U \subseteq F$ be a homogeneous submodule. Then, the module *F*/*U* is sequentially Cohen-Macaulay if and only if for all $i = 0,\ldots ,n$, we have ${\text {HF}}({\text {Ext}}^{n-i}_S(F/U,S)) = {\text {HF}}({\text {Ext}}^{n-i}_S(F/{\text {gin}}_{revlex} (U),S))$, where ${\text {HF}}(-)$ denotes the Hilbert function of a graded *S*-module.

This theorem has been extended in two different directions: weaker versions of sequential Cohen-Macaulayness can be characterized either by showing that equality of Hilbert functions holds only for certain cohomological indices [17], or by replacing revlex with certain partial revlex orders [5].

To state our main theorem, we recall the notion of arithmetic degree, introduced by Bayer and Mumford in [3] (see also [21, 22]). First, recall that if $0\ne M$ is a finitely generated $\mathbb {Z}$-graded module of dimension *d*, and ${\text {HF}}(M;j) = \dim _k(M_j)$ is the Hilbert function of *M* in degree *j*, then for $j \gg 0$, we have

$$\begin{aligned} \sum _{i \leqslant j} {\text {HF}}(M;i) = \frac{{\text {e}}(M)}{d!} j^{d} + O(j^{d-1}) \end{aligned}$$

for some ${\text {e}}(M) \in \mathbb {Z}_{>0}$, called multiplicity (or degree) of *M*. Given an integer $r \geqslant d$, we let

$$\begin{aligned} {\text {e}}_r(M) = {\left\{ \begin{array}{ll} {\text {e}}(M) &  \text { if } r=d, \\ 0 &  \text { otherwise. } \end{array}\right. } \end{aligned}$$

### Definition 1.2

Let $S=k[x_1,\ldots ,x_n]$, and *M* be a finitely generated graded *S*-module. For all $r=0,\ldots ,n$, we let ${\text {adeg}}_r(M) = {\text {e}}_r({\text {Ext}}^{n-r}_S(M,S))$. The arithmetic degree of *M* is defined as ${\text {adeg}}(M) = \sum _{r=0}^n {\text {adeg}}_r(M)$.

Recall that $\dim ({\text {Ext}}_S^{n-r}(M,S)) \leqslant r$ always holds (see for instance [18, Section 3.1]), therefore the above definition makes sense. Now let *F* be a free *S*-module, and $U \subseteq F$ be a graded submodule. Given any weight $\omega \in \mathbb {Z}^n$, by upper semi-continuity, one has ${\text {adeg}}_r(F/U) \leqslant {\text {adeg}}_r(F/{\text {in}}_\omega (U))$ for all $r=0,\ldots ,n$ (see [21] for the case of cyclic modules); in particular, ${\text {adeg}}(F/U) \leqslant {\text {adeg}}(F/{\text {gin}}_\textrm{revlex}(U))$ always holds. If *F*/*U* is sequentially Cohen-Macaulay, then Theorem 1.1 immediately gives that ${\text {adeg}}(F/U) = {\text {adeg}}(F/{\text {gin}}_\textrm{revlex}(U))$. This result was observed by Lu and Yu [16, Proposition 3.6], and in the same paper they conjecture that the converse holds as well. The purpose of this note is to prove their conjecture:

### Main theorem

Let $S=k[x_1,\ldots ,x_n]$, with the standard grading. Let *F* be a finitely generated graded free *S*-module and $U \subseteq F$ be a homogeneous submodule. We have that *F*/*U* is sequentially Cohen-Macaulay if and only if ${\text {adeg}}(F/U) = {\text {adeg}}(F/{\text {gin}}_\textrm{revlex}(U))$.

Actually, we prove their conjecture in a bigger generality, by showing that it is not necessary to take general coordinates when computing initial ideals as long as $x_n,\ldots ,x_1$ is a filter regular sequence for *F*/*U* (see Sect. 2). As a consequence of our proof, we also obtain an analogous generalization of Theorem 1.1.

## Preliminaries and main result

Let *k* be a field, and $S=k[x_1,\ldots ,x_n]$ be a polynomial ring with standard grading $\deg (x_i)=1$ for all *i*. Let $\mathfrak {m}=(x_1,\ldots ,x_n)$. Throughout, *M* will always denote a finitely generated $\mathbb {Z}$-graded *S*-module.

Consider the weight $\omega =(0,\ldots ,0,-1) \in \mathbb {Z}^n$ and, for a homogeneous submodule *U* of a graded free *S*-module *F*, we let ${\text {in}}(U):= {\text {in}}_\omega (U) \subseteq F$ be the initial submodule. Since revlex can be obtained as ${\text {in}}_\Omega$ for the following matrix of weights

$$\begin{aligned} \Omega = \begin{bmatrix} 0 &  \quad 0 & \quad \ldots &  \quad 0& \quad -1 \\ 0 &  & \quad 0 \ldots & \quad -1 & \quad -1 \\ \vdots &  \quad \vdots &  \quad \vdots & \quad \vdots & \quad \vdots \\ 0 & \quad -1 &  \quad \ldots & \quad -1 & \quad -1 \end{bmatrix}, \end{aligned}$$

of which $\omega$ is the first row, we call ${\text {in}}(U)$ a “partial revlex submodule”. See [5] for more details on this construction, where ${\text {in}}(-)$ is denoted as ${\text {in}}_{\textrm{rev}_{1}}(-)$.

### Definition 2.1

Let *M* be a finitely generated $\mathbb {Z}$-graded *S*-module. A homogeneous element $f \in S$ is called filter regular if $0:_M f$ has finite length. A sequence of elements $f_1,\ldots ,f_t$ is called a filter regular sequence if $f_{i+1}$ is filter regular for $M/(f_1,\ldots ,f_i)M$ for all $i<t$.

### Remark 2.2

Let *U* be a homogeneous submodule of a graded free *S*-module *F*. Since ${\text {in}}(U)$ is a partial deformation towards ${\text {in}}_\textrm{revlex}(U)$, one has that ${\text {adeg}}_r(F/U) \leqslant {\text {adeg}}_r(F/{\text {in}}(U)) \leqslant {\text {adeg}}_r(F/{\text {in}}_\textrm{revlex}(U))$ for all $r=0,\ldots ,n$ by upper semicontinuity.

In the proof of our main theorem, we will need the following lemma, which is an immediate consequence of a result of Serre.

### Lemma 2.3

Let *M* be a finitely generated $\mathbb {Z}$-graded *S*-module of dimension $d>0$, and let $\ell \in S_1$ be such that $M/\ell M$ is Cohen-Macaulay of dimension $d-1$, and ${\text {e}}_{d-1}(M/\ell M) = {\text {e}}_d(M)$. Then $\ell$ is an *M*-regular element, and *M* is Cohen-Macaulay.

### Proof

Without loss of generality, we may assume that *k* is infinite. Then, we can find linear forms $\ell _2,\ldots ,\ell _{d}$ which form a regular sequence for $M/\ell M$. Since $M/\ell M$ is Cohen-Macaulay, our assumptions guarantee that ${\text {e}}_d(M) = {\text {e}}_{d-1}(M/\ell M) = \lambda (M/(\ell ,\ell _2,\ldots ,\ell _d)M)$, where $\lambda (-)$ denotes the length of a module. By the graded version of [4, Theorem 4.7.10(b)], we conclude that *M* is Cohen-Macaulay and $\ell ,\ell _2,\ldots ,\ell _d$ is a regular sequence on *M*. $\square$

For a module *M*, we let ${\text {Ass}}_r(M) = {\text {Ass}}(M) \cap \{\mathfrak {p}\in {\text {Spec}}(S) \mid \dim (S/\mathfrak {p}) = r\}$.

### Remark 2.4

We recall that, if *M* is finitely generated and $\mathbb {Z}$-graded, then ${\text {Ass}}_r(M) = {\text {Ass}}_r({\text {Ext}}^{n-r}_S(M,S))$ for all $r = 0,\ldots ,n$. This follows by noticing that, for a prime $\mathfrak {p}$ such that $\dim (S/\mathfrak {p}) = r$, we have that $\mathfrak {p}\in {\text {Ass}}_r(M)$ if and only if $H^0_{\mathfrak {p}S_\mathfrak {p}}(M_\mathfrak {p}) \ne 0$, if and only if $\left( {\text {Ext}}^{n-r}_S(M,S)\right) _\mathfrak {p}\ne 0$. Since $\dim ({\text {Ext}}^{n-r}_S(M,S))\leqslant r$ (see for instance [18, Section 3.1]), this is equivalent to $\mathfrak {p}\in {\text {Min}}({\text {Supp}}({\text {Ext}}^{n-r}_S(M,S)))$, which in turn is equivalent to $\mathfrak {p}\in {\text {Ass}}_r({\text {Ext}}^{n-r}_S(M,S))$.

### Remark 2.5

Let *F* be a finitely generated free *S*-module, and $U \subseteq F$ be a homogeneous submodule. By [15, Theorem 2.2], we have that $F/{\text {gin}}_\textrm{revlex}(U)$ is sequentially Cohen-Macaulay. However, the same argument works for $F/{\text {in}}_\textrm{revlex}(U)$ assuming that $x_n,x_{n-1},\ldots ,x_1$ form a filter regular sequence for *F*/*U* (see also [5, Key example 2.15]). In particular, under these assumptions, for all $r=0,\ldots ,n$, the module ${\text {Ext}}^{n-r}_S(F/{\text {in}}_\textrm{revlex}(U),S)$ is either zero or Cohen-Macaulay of dimension *r*.

We are now ready to prove the conjecture of Lu and Yu [16].

### Theorem 2.6

Let $S=k[x_1,\ldots ,x_n]$, with the standard grading. Let *F* be a finitely generated graded free *S*-module and $U \subseteq F$ be a homogeneous submodule. Assume that $x_n,x_{n-1},\ldots ,x_1$ is a filter regular sequence for *F*/*U*. The following are equivalent:

1. *F*/*U* is sequentially Cohen-Macaulay,
2. $${\text {HF}}({\text {Ext}}^{n-r}_S(F/U,S)) = {\text {HF}}({\text {Ext}}^{n-r}_S(F/{\text {in}}_\textrm{revlex}(U),S))$$ for all $$r=0,\ldots ,n$$,
3. $${\text {adeg}}(F/U) = {\text {adeg}}(F/{\text {in}}_\textrm{revlex}(U))$$.

### Proof

We prove the equivalence of the three conditions by induction on $d=\dim (F/U)$. Let $V={\text {in}}_\textrm{revlex}(U)$. First of all, observe that $U^{{\text {{sat}}}} = U:x_n^\infty$, and that ${\text {in}}_\textrm{revlex}(U^{{\text {{sat}}}}) = V:x_n^\infty = V^{{\text {{sat}}}}$ by well-known properties of revlex-type orders (see [10, 15.7]). It follows that ${\text {HF}}(U^{{\text {{sat}}}}/U) = {\text {HF}}(V^{{\text {{sat}}}}/V)$. Since $U^{{\text {{sat}}}}/U$ is the graded Matlis dual of ${\text {Ext}}^n_S(F/U,S)$ (and similarly for *V*), we conclude that ${\text {HF}}({\text {Ext}}^n_S(F/U,S)) = {\text {HF}}({\text {Ext}}^n_S(F/V,S))$. In particular, ${\text {adeg}}_0(F/U) = {\text {e}}_0({\text {Ext}}^n_S(F/U,S)) = {\text {e}}_0({\text {Ext}}^n_S(F/V,S)) = {\text {adeg}}_0(F/V)$.

Since modules of dimension zero are automatically sequentially Cohen-Macaulay, for $d=0$, the three statements are trivially equivalent because they are all true.

Now assume that $d>0$. Recall that *F*/*U* is sequentially Cohen-Macaulay if and only if so is $F/U^{{\text {{sat}}}}$ [6, Corollary 2.8], and that ${\text {Ext}}^{n-r}_S(F/U,S) \cong {\text {Ext}}^{n-r}_S(F/U^{{\text {{sat}}}},S)$ for all $r>0$. Similar considerations hold for *V*. In view of the previous equalities, we may assume that ${\text {depth}}(F/U)>0$ after possibly replacing *U* with $U^{{\text {{sat}}}}$.

Assume (1). By Remark 2.4, we have ${\text {Ass}}_r(F/U)= {\text {Ass}}_r({\text {Ext}}^{n-r}_S(F/U,S))$. Also, ${\text {Ass}}_r({\text {Ext}}^{n-r}_S(F/U,S)) = {\text {Ass}}({\text {Ext}}^{n-r}_S(F/U,S))$ as ${\text {Ext}}^{n-r}_S(F/U,S)$ is either zero or Cohen-Macaulay of dimension *r*. Since $x_n$ is filter regular for *F*/*U*, we conclude that it is regular for ${\text {Ext}}^{n-r}_S(F/U,S)$ for all $r>0$. For all $r>0$, we have short exact sequenceswhich give that $F/(U+x_nF)$ is sequentially Cohen-Macaulay of dimension $d-1$. Moreover, for all $r>0$ and $j \in \mathbb {Z}$, we obtain

2.1 $$\begin{aligned}  &   {\text{ HF }}({\text{ Ext }}^{n-r+1}_S(F/(U+x_nF),S);j)\nonumber \\    &   \quad = {\text{ HF }}({\text{ Ext }}^{n-r}_S(F/U,S);j+1)- {\text{ HF }}({\text{ Ext }}^{n-r}_S(F/U,S);j). \end{aligned}$$

Now let $\overline{S} = k[x_1,\ldots ,x_{n-1}]$. We can identify $F/(U+x_nF)$ with a sequentially Cohen-Macaulay $\overline{S}$-module $\overline{F}/\overline{U}$, where $\overline{F}$ is a graded free $\overline{S}$-module and $\overline{U} \subseteq \overline{F}$ is a homogeneous submodule. Since $\dim (\overline{F}/\overline{U}) = d-1$, by induction, we have that ${\text {HF}}\left( {\text {Ext}}^{n-r}_{\overline{S}}(\overline{F}/\overline{U},\overline{S})\right) = {\text {HF}}\left( {\text {Ext}}^{n-r}_{\overline{S}}(\overline{F}/{\text {in}}_\textrm{revlex}(\overline{U}),\overline{S})\right)$ for all $r > 0$. By [4, Lemma 3.1.16], for all $r>0$, we have that

$$\begin{aligned} {\text {Ext}}^{n-r}_{\overline{S}}(\overline{F}/\overline{U},\overline{S})\cong {\text {Ext}}^{n-r+1}_S(F/(U+x_nF),S) \end{aligned}$$

and

$$\begin{aligned} {\text {Ext}}^{n-r}_{\overline{S}}(\overline{F}/{\text {in}}_\textrm{revlex}(\overline{U}),\overline{S})\cong {\text {Ext}}^{n-r+1}_S(F/(V+x_nF),S). \end{aligned}$$

Using that ${\text {in}}_\textrm{revlex}(U+x_nF) = V+x_nF$ (see [10, 15.7]), we obtain

$$\begin{aligned}  &   {\text {HF}}({\text {Ext}}^{n-r+1}_S(F/(U+x_nF),S)) \\  &   \quad = {\text {HF}}({\text {Ext}}^{n-r+1}_S(F/{\text {in}}_\textrm{revlex}(U+x_nF),S)) \text { for all } r>0. \end{aligned}$$

By the proof of [5, Proposition 3.5] and by [5, Lemma 2.9], for all $r>0$, we have short exact sequences2.2which give

2.3 $$\begin{aligned}  &   {\text {HF}}({\text {Ext}}^{n-r+1}_S(F/V+x_nF,S);j) \nonumber \\  &   \quad = {\text {HF}}({\text {Ext}}^{n-r}_S(F/VS);j+1) - {\text {HF}}({\text {Ext}}^{n-r}_S(F/V,S);j). \end{aligned}$$

Combining (2.1) and (2.3), together with the fact that the ${\text {Ext}}$ modules vanish for sufficiently negative degrees, we finally obtain

$$\begin{aligned} {\text {HF}}({\text {Ext}}^{n-r}_S(F/U,S)) = {\text {HF}}({\text {Ext}}^{n-r}_S(F/V,S)) \text { for all } r>0, \end{aligned}$$

and this concludes the proof that (1) $\Rightarrow$ (2). The fact that (2) $\Rightarrow$ (3) is trivial.

We finally show that (3) $\Rightarrow$ (1). Let $\omega =(0,\ldots ,0,-1) \in \mathbb {Z}^n$, and let $W={\text {in}}_\omega (U)$. By Remark 2.2 and our assumptions, we must have ${\text {adeg}}_r(F/U)={\text {adeg}}_r(F/W) = {\text {adeg}}_r(F/V)$ for all $r=0,\ldots ,n$. Again using the proof of [5, Proposition 3.5] and [5, Lemma 2.9], for all $r>0$, we obtain short exact sequences2.4where we use that $W+x_nF = U+x_nF$ holds because $\omega$ is a partial revlex order. The short exact sequences (2.4) and (2.2) give that ${\text {adeg}}_{r-1}(F/(U+x_nF)) = {\text {adeg}}_r(F/W) = {\text {adeg}}_r(F/V) = {\text {adeg}}_{r-1}(F/(V+x_nF))$. We therefore obtain that ${\text {adeg}}(F/(U+x_nF)) = {\text {adeg}}(F/(V+x_nF))$. As above, we can identify $F/(U+x_nF)$ with an $\overline{S}$-module $\overline{F}/\overline{U}$. In this way, $F/(V+x_nF)$ can be identified with $\overline{F}/{\text {in}}_\textrm{revlex}(\overline{U})$, and therefore we have that ${\text {adeg}}(\overline{F}/\overline{U}) = {\text {adeg}}(\overline{F}/{\text {in}}_\textrm{revlex}(\overline{U}))$. Since $\dim (\overline{F}/\overline{U})=d-1$ and $x_{n-1},\ldots ,x_1$ form a filter regular sequence for $\overline{F}/\overline{U}$, by induction, we have that $\overline{F}/\overline{U}$ is sequentially Cohen-Macaulay, and so is $F/(U+x_nF)$.

Now we note that if ${\text {Ext}}^{n-r}_S(F/U,S) \ne 0$, then ${\text {Ext}}^{n-r}_S(F/V,S) \ne 0$ by upper semi-continuity. Since we have ${\text {e}}_r({\text {Ext}}^{n-r}_S(F/U,S)) = {\text {adeg}}_r(F/U) = {\text {adeg}}_r(F/V) = {\text {e}}_r({\text {Ext}}^{n-r}_S(F/V,S))$, and the latter is positive by Remark 2.5, it follows that $\dim ({\text {Ext}}^{n-r}_S(F/U,S))=r$. To complete the proof, we show by induction on $r>0$ that if ${\text {Ext}}^{n-r}_S(F/U,S) \ne 0$, then it is a Cohen-Macaulay module, and that $x_n$ is a regular element for it.

First note that for $r>0$, since ${\text {Ass}}_r(F/U) = {\text {Ass}}_r({\text {Ext}}^{n-r}_S(F/U,S))$ by Remark 2.4, and because $x_n$ is filter regular for *F*/*U*, we have that $x_n$ avoids all minimal primes of ${\text {Supp}}({\text {Ext}}^{n-r}_S(F/U,S))$ of dimension *r*. In particular, $\dim ({\text {Ext}}^{n-r}_S(F/U,S) \otimes _S S/(x_n)) = r-1$.

For $r=1$, we have an exact sequencewhich gives that ${\text {Ext}}^n_S(F/(U+x_nF),S) \cong {\text {Ext}}^{n-1}_S(F/U,S) \otimes _S S/(x_n)$. Using the short exact sequence (2.4), and recalling that $U+x_nF = W + x_nF$, we obtain

$$\begin{aligned} {\text {e}}_1({\text {Ext}}^{n-1}_S(F/U,S))&= {\text {e}}_1({\text {Ext}}^{n-1}_S(F/W,S)) \\&= {\text {e}}_0({\text {Ext}}^n_S(F/(U+x_nF),S))\\&= {\text {e}}_0({\text {Ext}}^{n-1}_S(F/U,S) \otimes _S S/(x_n)). \end{aligned}$$

By Lemma 2.3, we conclude that ${\text {Ext}}^{n-1}_S(F/U,S)$ is Cohen-Macaulay, and $x_n$ is regular for it.

Now assume that ${\text {Ext}}^{n-i}_S(F/U,S)$ is Cohen-Macaulay for all $i=1,\ldots ,r-1$ and that $x_n$ is a regular element for all such modules. In particular, it is regular for ${\text {Ext}}^{n-r+1}_S(F/U,S)$, and therefore we have an exact sequenceWe conclude that ${\text {Ext}}^{n-r+1}_S(F/(U+x_nF),S) \cong {\text {Ext}}^{n-r}(F/U,S) \otimes _S S/(x_n)$. As before, we have

$$\begin{aligned} {\text {e}}_r({\text {Ext}}^{n-r}_S(F/U,S))&= {\text {e}}_r({\text {Ext}}^{n-r}_S(F/W,S)) \\&= {\text {e}}_{r-1}({\text {Ext}}^{n-r+1}_S(F/(U+x_nF),S)) \\&= {\text {e}}_{r-1}({\text {Ext}}^{n-r}_S(F/U,S) \otimes _S S/(x_n)). \end{aligned}$$

Finally, by Lemma 2.3, we conclude that ${\text {Ext}}^{n-r}_S(F/U,S)$ is Cohen-Macaulay and $x_n$ is regular for it, and the proof is complete. $\square$

### Remark 2.7

We would like to point out that, if the field *k* is infinite, then the assumption that $x_n,\ldots ,x_1$ form a filter regular sequence on *F*/*U* is not at all restrictive. In fact, by prime avoidance, we can always find a sequence of linearly independent linear forms $\ell _n,\ldots ,\ell _1$ which form a filter regular sequence on *F*/*U*. After performing the change of coordinates $x_i:= \ell _i$ for $i=1,\ldots ,n$, we are in the assumptions of Theorem 2.6.
